# Effect of *Rhizoma Drynariae* on differential gene expression in ovariectomized rats with osteoporosis based on transcriptome sequencing

**DOI:** 10.3389/fendo.2022.930912

**Published:** 2022-08-02

**Authors:** Hui Su, Haipeng Xue, Shang Gao, Binghan Yan, Ruochong Wang, Guoqing Tan, Zhanwang Xu, Lingfeng Zeng

**Affiliations:** ^1^ First Clinical Medical College, Shandong University of Traditional Chinese Medicine, Jinan, China; ^2^ Affiliated Hospital of Shandong University of Traditional Chinese Medicine, Jinan, China; ^3^ College of traditional Chinese medicine, Beijing University of Traditional Chinese Medicine, Beijing, China; ^4^ The 2nd Affiliated Hospital of Guangzhou University of Chinese Medicine, Guangzhou, China

**Keywords:** *Rhizoma Drynariae*, ovariectomized, rats, osteoporosis, transcriptome sequencing

## Abstract

Osteoporosis is increasingly becoming a serious problem affecting the quality of life of the older population. Several experimental studies have shown that Chinese medicine has a definite effect on improving osteoporosis. Based on transcriptome sequencing, we analyzed the differential gene expression and mechanism of the related signaling pathways. Fifteen rats were randomly divided into an experimental group, a model group, and a sham surgery group. The rat model for menopausal osteoporosis was established using an ovariectomy method. One week after modeling, the experimental group was administered(intragastric administration)8.1 g/kg of *Rhizoma drynariae*, whereas the model and sham groups received 0.9% saline solution twice daily for 12 weeks. Subsequently, the rats were sacrificed, and the left femur of each group was removed for computerized tomography testing, while right femurs were used for hematoxylin and eosin staining. High-throughput RNA sequencing and functional and pathway enrichment analyses were performed. Comparing the gene expression between the experimental and model groups, 149 differential genes were identified, of which 44 were downregulated and 105 were upregulated. The criteria for statistical significance were |log2 Fold Change| > 1 and *P* < 0.05. Gene ontology analysis showed that the differentially expressed genes were enriched in cell component terms such as cell part and outer cell membrane part, and the genes were associated with cell process, biological regulation, metabolic processes, DNA transcription, and catalytic activity. Enrichment analysis of Kyoto Encyclopedia of Genes and Genomes pathways showed significantly enriched pathways associated with systemic lupus erythematosus, herpes simplex infection, circadian rhythm, vascular smooth muscle contraction, the AGE-RAGE signaling pathway in diabetic complications, and the TNF, Apelin, and Ras signaling pathways. Our results revealed that the Npas2, Dbp, Rt1, Arntl, Grem2, H2bc9, LOC501233, Pla2g2c, Hpgd, Pde6c, and Dner genes, and the circadian rhythm, lipid metabolism, inflammatory signaling pathway, and immune pathways may be the key targets and pathways for traditional Chinese medicine therapy of Rhizoma Drynariae in osteoporosis.

## 1 Introduction

Osteoporosis, a disease with increased bone fragility and risk of fracture due to decreased bone formation and increased destruction of bone resorption (1, 2),is progressively becoming a serious problem affecting the quality of life of the older population.

In the study of the pathophysiology of osteoporosis, we have found that factors associated with bone strength and damage include a weakened microstructure and tissue of the trabecular bone and decreased bone formation with excessive bone resorption. These factors are closely related to age and changes in estrogen levels (3).In terms of bone metabolism, the pathogenesis of osteoporosis lies in the imbalance between bone resorption and bone formation.

Current drug treatments for osteoporosis include basic supplements (mainly calcium and vitamin D), bone absorption inhibitors [such as bisphosphonates, calcitonins, and selective estrogen receptor modulators (4)];and bone formation promoters [including thyroxacine (5)]. Additionally, treatment with strontium ranelate has a bidirectional effect, i.e., inhibiting bone resorption and promoting bone formation (6).

Postmenopausal osteoporosis, also known as primary osteoporosis, is common in older, postmenopausal women, presenting bone pain, adverse limb movement, and an increased risk of bone fracture. Estrogen deficiency is the major cause of postmenopausal bone loss, while older age, smoking, chronic disease, and other related illnesses and drugs are additional causes (7, 8).

Chinese herbal medicine has an apparent effect on osteoporosis, as it can ameliorate bone health through multiple targets and pathways (9).Rhizoma Drynariae is a traditional Chinese medicine that improves bone by promoting trauma recovery (10). Although there are some studies on the effect of bone repair in osteoporosis (11, 12) (13), the mechanisms and targets are difficult to elucidate due to their complex composition (14, 15). Here, we aim to explore the specific action of target genes using transcriptome sequencing technology and reveal the molecular mechanisms related to the efficacy of Rhizoma Drynariae. The identification of these mechanisms will promote the use of Chinese herbal medicine in the clinical treatment of osteoporosis.

## 2 Materials and methods

### 2.1 Animals and medicines

Female Sprague-Dawley rats (6-weeks-old, weight 200–220 g) were purchased from Beijing Vital River Laboratory Animal Technology Co., Ltd. The feeding environment was clean and well ventilated, and water was freely available. All animals were housed at the SPF Grade Animal Experimental Center of Shandong Provincial Hospital of Traditional Chinese Medicine at 25°C with a constant humidity of 50% for 12 h per day and given a standard diet. The experimental animals were bred for 7 d, and 15 rats were divided into the experimental group (OVXDF),the model group (OVX), and the sham operation group (SHAM). In the OVXDF and OVX groups, a preoperative diet was not provided for 12 h, and intraperitoneal pentobarbital sodium (10g/L) was used for anesthesia. The OVXDF group was subjected to surgical resection of their bilateral ovaries, and only small peripheral adipose tissue around the ovary was removed in the SHAM group (16). After the operation, an intramuscular injection of penicillin (50,000 U/d) was administered to prevent infection. On the seventh day after the operation, we administered Rhizoma Drynariae to the rats by intragastric administration.

The preparation of drug decoction was followed the preliminary preparation protocol of our research group (17, 18), Granules of Rhizoma Drynariae was purchased from the pharmacy of the Affiliated Hospital of Shandong University of Traditional Chinese Medicine. Next, 100 g of Rhizoma Drynariae was placed in a pot distilled with water and concentrated to 100 mL. Liquid Rhizoma Drynariae (1 g/mL) was obtained and stored at 4°C.

Following the animal dose conversion formula (19), Rhizoma Drynariae gavage was administered at a potency of 8.1 g/kg to the OVXDF group, and 0.9% saline solution was administered to the OVX and SHAM groups twice daily for 12 weeks. The experimental procedures were conducted in accordance with the management and use of experimental animal guidelines of the Shandong Provincial Hospital of Traditional Chinese Medicine, and the study was approved by the ethical review committee (Affiliate hospital of Shandong University of Traditional Chinese Medicine 2021-26).

### 2.2 Bone mineral density measurements and micro-CT of bone

Rats were sacrificed following a sodium pentobarbital overdose(60mg/kg) (20), and bilateral femurs were removed. The left femurs were subjected to computerized tomography (CT) testing with a tone-beam bench-top, miniature CT device (μCT80, Scanco Medical, Brüttisellen, Switzerland), and images were analyzed using μCT80 Evaluation Program v6.51 (Scanco Medical). Pellellous bones with the distal femur 1 mm above the growth plate were selected after scanning a volume-of-interest restricted to the internal femur region. The trabecular and cortical bones were extracted by drawing free-form contour lines using the CT analyzer software. Using the microstructure of cancellous bone, 3D images were obtained by multiplane reorganization. The region of interest was analyzed using the CT Analyser v1.18.8.0 (Bruker, Germany). Using the uniform parameters, bone mineral density (BMD; g/cm3), bone volume fraction [BV/total volume (TV); %], bone surface area volume fraction (BS/TV; 1/mm), and the number of trabecular bone (Tb.N; 1/mm) were calculated.

### 2.3 Hematoxylin and eosin staining and TRAP staining

#### 2.3.1 HE staining

The right femur of each group was taken, cleaned and fixed in 10% formaldehyde solution for 48 h, washed in running water and distilled water for 24 h. The specimen was decalcified, and the decalcification was stopped after 3~4 h. The alkaline solution was washed for 12h, then dehydrated, transparent and wax, respectively, embedded and sliced in paraffin, and the drying time was about 10~15 min.

#### 2.3.2 TRAP staining

The right femoral paraffin sections of each group were dewaxed for 5 to 10 min for 5min with absolute ethanol, 90% ethanol, and 70% ethanol for 2min each. After washing and drying naturally for 2min, the fixative was fixed for 30s~3min at room temperature, stained with appropriate staining solution, covering the tissue, placed in a 37°C temperature box, and washed for 45~60min. Compound staining was performed for 5 to 8 min or methyl green staining for 2 to 3 min. Finally, it was washed, dried and micro examine.

### 2.4 High-throughput RNA sequencing

Total RNA was extracted from the bone tissue samples selected from each group after using TRIzol (Invitrogen, CA, USA), Samples are sequenced on the platform to get image files, which are transformed by the software of the sequencing platform, and the original data is generated. we use Cutadapt (v1.15) software to filter the sequencing data to get high quality sequence for further analysis.Finally obtained the expression of differential genes between the OVX group and OVXDF group.

### 2.5 Differential expression analysis and hierarchical clustering

Differential gene expression was analyzed using DESeq (R package). Differentially expressed genes (DEGs) were selected with |log2 Fold Change| > 1 and *P* < 0.05 as criteria for statistical significance. DEGs were represented using graphs to calculate the number of upregulated and downregulated DEGs in each comparison group. Volcano maps showing gene distribution of the DEGs were obtained using ggplot2 (R package). Two-way clustering analysis of the union and samples of DEGs across all comparison groups was performed using Pheatmap (R package).

### 2.6 Functional and pathway enrichment analysis

Gene ontology (GO) enrichment analysis using topGO (*P* < 0.05) was performed to determine the significantly enriched GO terms for DEGs and identify their main biological functions. Kyoto Encyclopedia of Genes and Genomes (KEGG) pathway enrichment analysis was performed using the clusterProfiler v3.4.4 software, focusing on significantly enriched pathways with *P* < 0.05.

### 2.7 Protein-protein interaction analysis

Protein interaction analysis was performed employing the Search Tool for the Retrieval of Interacting Genes (STRING) database (https://string-db.org/) to reveal the functional relationship between the target genes. When PPI information for this species was included in the STRING database, PPI action pairs containing DEGs and scores > 0.95 were screened based on the results of the DEG analysis. Differential gene correlation was also assessed by visual analysis using the Cytoscape v3.7.2 software.

### 2.8 Validation of RNA-seq results using qRT-PCR

Bone tissue was taken 50 mg to 1 mL Trizol using liquid nitrogen, 0.2 mL of chloroform, total RNA was extracted by Trizol, RNA concentration, purity and integrity, and cDNA. The corresponding PCR reaction amplification system (25 L) was added, PCR thermocycler amplification, amplification conditions: 95°C pre-denaturation for 3 min; 95°C was denatured for 20s, 60°C annealed for 15s, 72°C extended for 30s, and amplified in 45 cycles. The last 72°C was extended for 1 min. For SDS polyacrylamide gel electrophoresis, the image analysis software analyzed the band density, and the relative expression of the genes was calculated using the 2-△△Ct method. Five DEGs with different expression patterns were randomly chosen to validate the accuracy of the transcriptome sequencing results, qRT-PCR was verified using actin as the internal reference. The primers used are listed below (**Table 1**).

**Table 1:** Primers used for RT-qPCR test.

Gene	Forward Primer	Reverse Primer
Grem2	CCTGAAGAGTGACTGGTGCAAGAC	GTGTCGTGGGATGTAGAAGGAGTTG
Dbp	CACCGCTTCTCAGAGGAGGAATTG	CCTCTTGGCTGCTTCATTGTTCTTG
Dner	TGGAGCCCTGTGTGTAGCCTTC	CGTTGCTGAACTCACTGTCAATGC
Arnt1	AACCATTGTCCAGCCGTCATCTTC	AACCATTGTCCAGCCGTCATCTTC
npas2	GCTGATGTTGGAGGCGTTAGATGG	ATGTCCGAGGAGAGGCGTGATG
GAPDH	GAACATCATCCCTGCATCCA	GCCAGTGAGCTTCCCGTTCA

### 2.9 Statistical analysis

All the data were expressed as the mean ± standard deviation. An independent sample t-test evaluated the significant differences between two groups using SPSS v24.0 (SPSS, IL, USA), and *P* < 0.05 was considered significant. GraphPad Prism v7.0 (GraphPad Software, CA, USA) was utilized. All assays were conducted three times or more.

## 3.Results

### 3.1 Rat bone density measurement and micro-CT

**Figure 1:**
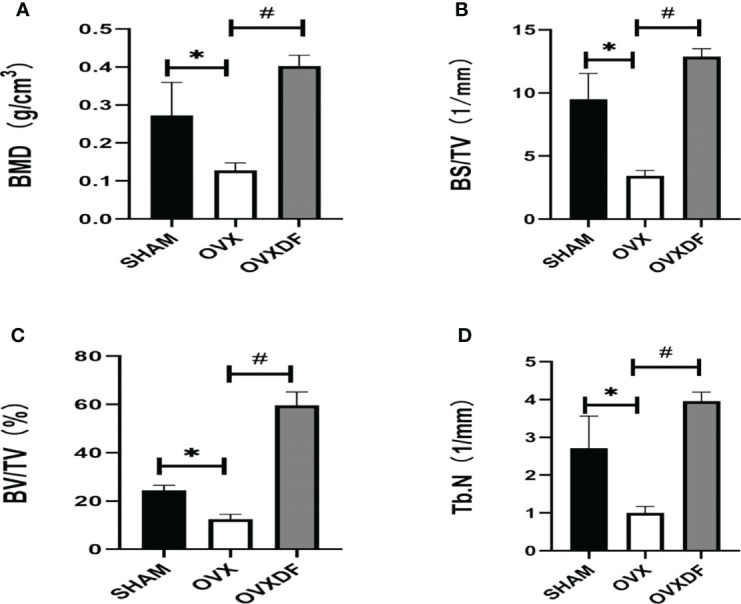
Bone parameter values of Micro-CT. **(A)** The BMD indicates the bone mineral density in the bone tissue of the region of interest. **(B)** Bone surface area and tissue volume ratio (BS/TV) can indirectly reflect the amount of bone mass. **(C)** Bone body and fraction (BV/TV) are commonly used in the evaluation of cortical and cancellous bone mass, **(D)** Number of trabecular bone (Tb.N) to evaluate the spatial morphological structure of trabecular bone. *P<0.05 versus SHAM group, ^#^ *P*<0.05, versus OVXDF group, n = 3 per group, Data were shoen as mean ± sd.

The results showed that the bone mineral density of the OVX group was significantly lower than the SHAM group (*P* < 0.05). In contrast, the bone mineral density of the OVXDF group was significantly higher than that of the OVX group (*P* < 0.05). Moreover, the bone volume score (BV/TV), surface area volume fraction (BS/TV), and number of trabecular bones (Tb.N) for the OVXDF and SHAM groups were significantly higher than those of the OVX group (*P* < 0.05; **Figure 1**).

**Figure 2:**
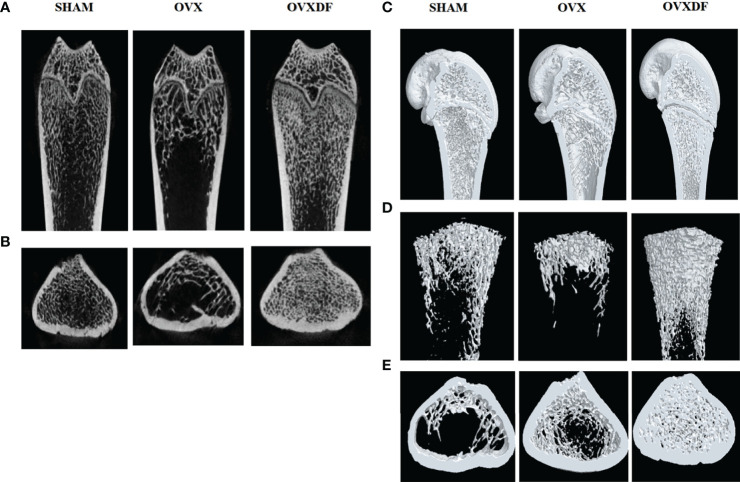
Micro-CT images of the femur. **(A, B)** 2D images in three groups of the distal femur (scale bars, 1 mm). **(C–E)** 3D images in three groups of the proximal femur (scale bars, 1 mm).

Micro-CT images showed that the trabecular bone in the OVX group had a sparse microstructure. However, in the OVXDF group, the trabecular bone is large, with dense arrangement and small gaps, indicating that the Rhizome Drynaria improves the trabecular microstructure of the femur (**Figure 2**).

### 3.2 H&E staining and TRAP staining

**Figure 3:**
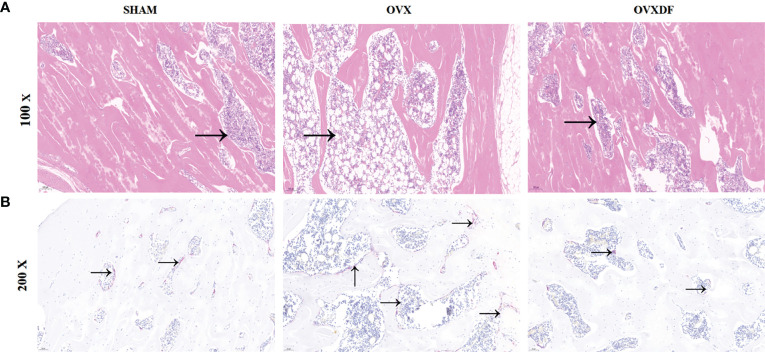
H&E staining images of femur bone: **(A)** H&E staining: Scale bar = 100mm, 100×; →= bone resorption holes; **(B)** TRAP staining: Scale bar = 500mm, 200×. →=osteoclast.

The femurs in the OVX group were thinning and decreasing, with apparent osteoporosis characteristics such as fractures and increased bone resorption holes. Rats in the SHAM group had abundant cancellous bone, many bony trabeculae, a tight arrangement, complete bone tissue structure, and a thick and uniform wall. In the OVXDF group, the structure of the femoral trabecular bone was relatively complete, the texture was tightly arranged, the bone trabecular connectivity was better, and the wall thickness was denser compared with the OVX group. Osteoclasts were stained red in TRAP staining, and the staining result showed that compared with the SHAM group, the OVX group osteoclasts were more numerous and widely distributed, while compared with the OVX group, the OVXDF group osteoclasts were fewer and concentrated. Overall, the state of the trabecular and cancellous bones in the OVXDF group was superior to that of the OVX group (**Figure 3**).

### 3.3 High-throughput RNA sequencing

**Figure 4:**
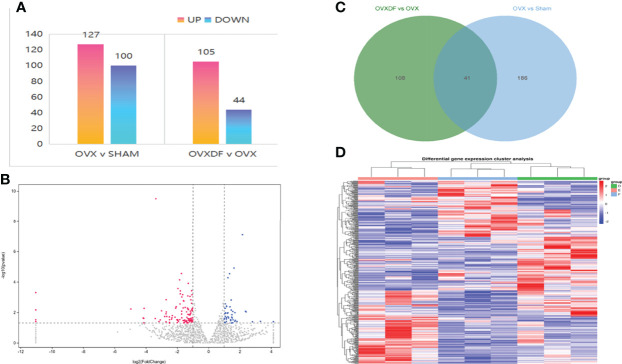
the comparison of gene expression in the SHAM group and the OVX group. **(A)** column diagram of DEGs : The abscissa represents the comparison group for the difference analysis, the ordinate indicates the number of difference genes, the color indicates regulation. Blue=up, orange=down. **(B)** Volcano plot of DEGs: The abscissa is log2FoldChange and the ordinate is the significance level for the negative log value of 10. The two vertical dashed lines in the figure are the threshold of expression difference multiple; the horizontal dashed line is the significance level threshold. Colors indicate that genes are up, downregulated, or not significantly differentially expressed. **(C)** Venn diagram of DEGs: The sum of the numbers in each circle represents the total number of DEGs for the comparison combination, and the overlapping part of the circle represents the DEGs shared between the two comparison groups. **(D)** heat map of DEGs: The horizontal lines represents genes, each column is a sample, red represents high-expressed genes, and blue represents low-expressed genes. D=OVXDF E=OVX F=SHAM.

A total of 17,591 transcriptome mRNA was obtained from the OVXDF and OVX groups. Based on the results of the differential gene analysis, 416 DEGs were identified in the OVXDF, OVX, and SHAM groups, and the mutual relationship was displayed in a Venn diagram (**Figure 4**). In comparing gene expression between the OVXDF and OVX groups, 149 DEGs were identified, of which 44 were down regulated and 105 were upregulated. Further screening of the most significant gene expression showed 12 DEGs, including 8 upregulated and 4 downregulated genes (**Table 2**). Moreover, we performed a two-way clustering analysis of the acquired genes between the three groups (**Figure 4**). In comparing gene expression between the SHAM and OVX groups, 227 DEGs were identified, of which 110 were upregulated and 127 were downregulated. These details were presented as column diagrams and volcano charts (**Figure 4**).

**Table 2:** 12 significantly DEGs in OVXDF group and OVX group.

Name	log2FoldChange	pval	Regulation
Npas2	3.37324286	3.16E-10	up Regulation
Dbp	-2.177429033	7.93E-08	down Regulation
LOC103690108	11.03293695	0.000499027	up Regulation
Arntl	2.702206184	0.001462716	up Regulation
Grem2	-2.060425268	0.004194338	down Regulation
H2bc9	2.094632641	0.005218957	up Regulation
LOC501233	4.110331215	0.00549491	up Regulation
Pla2g2c	4.958704937	0.005965812	up Regulation
Hpgd	2.998655062	0.007817667	up Regulation
Pde6c	-2.366381804	0.008314784	down Regulation
AABR07065750.2	-2.405047687	0.009304366	down Regulation
Dner	2.691923189	0.009380164	up Regulation

| log2FoldChange |> 2 with significant P-value <0.01.

### 3.4 Functional and pathway enrichment analysis

#### 3.4.1 GO enrichment analysis of the DEGs

**Figure 5:**
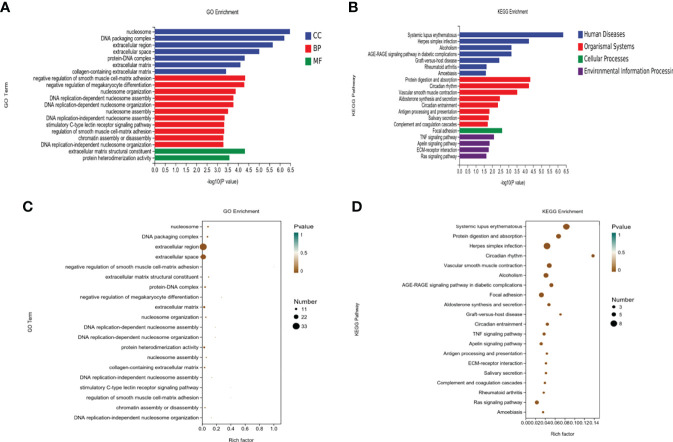
Enrichment analysis of the DEGs. **(A, B)** Top 20 of KEGG pathway analysis of DEGs. **(C, D)** Top 20 of Gene Ontology (GO) analysis of DEGs including biological process (BP), cell component (CC), and molecular function (MF).

Enrichment analysis of DEGs (**Figure 5**) from the OVXDF and OVX groups showed that 149 DEGs were collectively enriched in 362 molecular functions, 253 cellular components, and 2478 biological processes. Considering *P* < 0.05 as the criterion for significant enrichment, the top enrichment scores were ranked in cell part, outer cell membrane part, cell process, biological regulation, metabolic process, DNA transcription, and catalytic activity. DEGs enriched in molecular function included extracellular matrix (ECM) structural constituent and protein heterodimerization activity. DEGs enriched in cellular component terms, facilitated by the major enrichment in the nucleosome, included DNA and protein-DNA packaging complex, extracellular space, and collagen-containing ECM. Furthermore, the DEGs were involved in biological processes such as negative regulation of smooth muscle cell-matrix adhesion, negative regulation of megakaryocyte differentiation, nucleosome organization, DNA replication-dependent and -independent nucleosome assembly, stimulatory C-type lectin receptor signaling pathway, and chromatin assembly or disassembly. Regulation of smooth muscle and cell-matrix adhesion contribute a large proportion of the biological process classification and changes in both states are closely related to the aging process. Whether the appearance of vascular calcification or cell-matrix adhesion affects the Wnt signaling pathway, they all cause bone loss to some extent. The outer part of the cell membrane accounted for the most proportion of DEGs in the cell group classification. In the molecular function classification, ECM structural constituent, mainly by protein heterodimerization activity, can promote stem cell proliferation and maturation, the formation of matrix scaffold, and bone remodeling by bone-forming cells. It has been shown that[] changes in the functional properties of the ECM may be involved in diseases such as osteoporosis and oral bone loss.

#### 3.4.2 KEGG enrichment analysis

DEGs from the OVXDF and OVX groups were mapped to the KEGG pathway database for enrichment analysis (**Figure 5**), obtaining 165 signaling pathways (*P* < 0.05), with 110 downregulated and 118upregulated pathways. Top-scoring pathways included systemic lupus erythematosus, herpes simplex infection, alcoholism, the AGE-RAGE signaling pathway in diabetic complications, and graft-versus-host disease. The diseases associated with the DEGs included rheumatoid arthritis, amoebiasis, and organismal systems. Moreover, the DEGs were strongly correlated with protein digestion and absorption, circadian rhythm, vascular smooth muscle contraction, aldosterone synthesis and secretion, antigen processing and presentation, salivary secretion, complement and coagulation cascades, and cellular processes. The most enrichment was in focal adhesion and environmental information processing. Furthermore, the DEGs were primarily enriched in inflammatory immune signaling pathways such as the TNF, Apelin, and Ras signaling pathways and ECM-receptor interaction.

#### 3.4.3 PPI network related to DEGs

**Figure 6:**
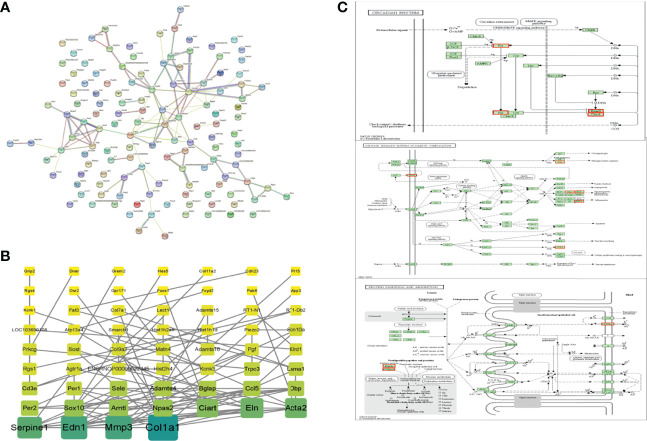
PPI networks of DEPs. **(A)** STRING data base (https://string-db.org/), Network nodes represents proteins, Edges represents protein-protein associations **(B)** Cytoscape3.7.2, Color and rectangular area are proportional to correlation and the darker color, the larger rectangular area and the greater correlation of DEPs. **(C)** top-ranked KEGG pathway based on the association degree analysis of DEPs. DEPs were marked in red. \Circadian rhythm.\Protein digestion and absorption. \AGE-RAGE signaling pathway in diabetic \complications.

DEGs from the OVXDF group were analyzed using the STRING data (https://string-db.org/) and imported into Cytoscape v3.7.2. The results showed 130 nodes and 111 edges, with an average node degree of 1.71. Genes with a large correlation between them were Col1a1 (collagen type I alpha 1 chain; degree = 12), Edn1 (endothelin 1; degree = 10), Mmp3 (matrix metallopeptidase 3; degree = 10), Serpine1 (serpin family E member 1; degree = 9), Acta2 (actin alpha 2; degree = 8), Eln (elastin; degree = 8), Ciart (circadian associated repressor of transcription; degree = 7), Arntl (aryl hydrocarbon receptor nuclear translocator-like; degree = 6), Npas2 (neuronal PAS domain protein 2; degree = 6), and Sox10 (SRY-box transcription factor 10; degree = 6) (**Figure 6**). Related genes were mostly enriched in the circadian rhythm, protein digestion and absorption, and the AGE-RAGE signaling pathway in diabetic complications (**Figure 6**).

### 3.5 Results of qRT-PCR

**Figure 7:**
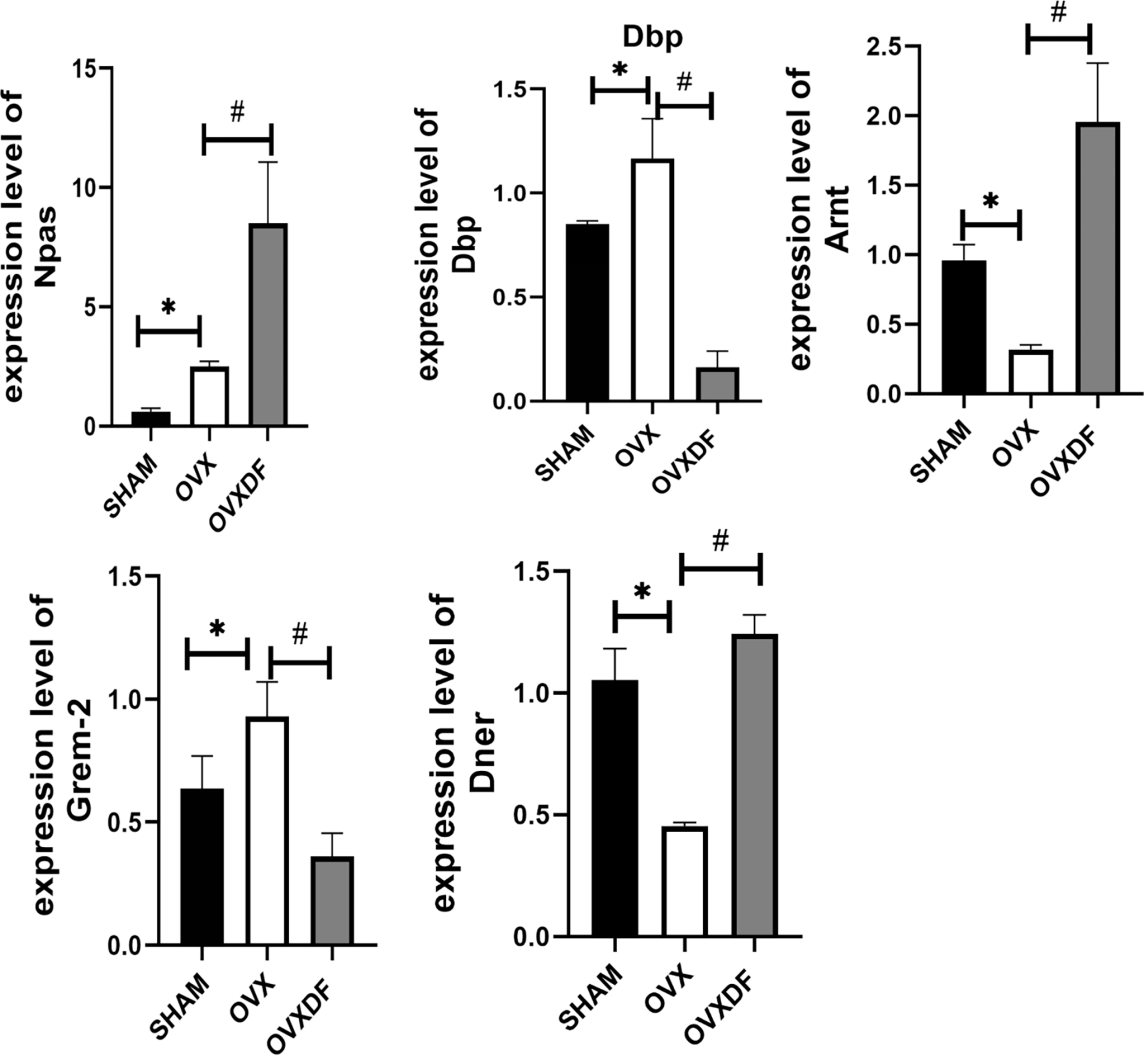
RT-qPCR results indicated that expression level of Npas2, Dbp,Arntl, Grem2 and Dner. *P<0.05 versus sham group, ^#^P<0.05, versus OVX groups,n = 3 per group, Data were shown as mean ± SD.

Following our transcriptomic sequencing results, five DEGs with apparent upregulation and downregulation were selected as qRT-PCR validation targets, namely Npas2, Dbp, Arntl, Grem2, and Dner. Dbp was significantly upregulated in the OVXDF group compared with that in the OVX group. However, the expression of Npas2, Arntl, Grem2, and Dner was significantly downregulated. These results were consistent with our RNA-seq results, thus reaffirming them (**Figure 7**).

## 4. Discussion

In this study, we aimed to identify DEGs and their expression patterns for promoting bone repair in osteoporosis and further reveal the specific mechanism of genes in osteoporosis treatment through transcriptomic analysis of osteoporosis model rats (OVX) and experimentally treated osteoporosis rats (OVXDF). A total of 149 DEGs were identified between the two groups. The 10 most altered DEGs were Npas2, Dbp, RT1, Arntl, Grem2,H2bc9, LOC501233, Pla2g2c, Hpgd, Pde6c, and AABR07065750. these may be a potential target for treatment in ovariectomized rats with osteoporosis. We confirmed the positive role of Rhizoma Drynariae in improving osteoporosis by an experimental analysis of rat femoral tissue.

Combined with the results of the DEG analysis, we found that DEGs involved in Rhizoma Drynariae regulation are mainly related to the circadian rhythm, lipid metabolism-related diseases, osteoporosis disease, inflammation, and tumors. Osteoporosis treatment with Rhizoma Drynariae can play a significant role through multiple pathways, and we confirmed that the Ras signaling pathway is important to regulate bone homeostasis and bone formation (21). Ras signaling combines with the downstream ERK signaling to promote osteoblast differentiation and bone formation during early skeletal development (22).The study by Peng (23) used miR-483-5p to down regulate Ras signaling by inhibiting RPL31 expression, resulting in the inhibition of osteogenic differentiation. Furthermore. Chen et al. (24) showed that the Apelin signaling pathway can activate mitophagy in bone marrow-derived mesenchymal stem cells, improve oxidative stress, and promote osteogenic function. One study (25) demonstrated that the Wnt/β-catenin pathway may be the link between Apelin-13 and bone mineral density, but its precise role in bone metabolism is currently unknown.

The mechanisms of the inflammatory signaling and immune pathways in osteoporosis have been widely studied. Our analysis confirmed that Rhizoma Drynariae could regulate the tumor inflammatory pathway by changing the levels of inflammatory factors and reducing the inflammatory response to regulate the immune status. The prostaglandin dehydrogenase gene Hpgd also plays a role in tumor development and inflammation (26). In our enrichment analysis, differential genes, including H2bc9 and AABR07065750.2, were mostly associated with immune system diseases such as systemic lupus erythematosus, rheumatoid arthritis, and herpes simplex infection; the release of inflammatory cytokines and autoantibody production during the disease development leads to local and systemic bone loss (27). The use of drugs such as glucocorticoids in their treatment also exacerbates the risk of osteoporosis. Moreover, the National Institutes of Health has identified graft-versus-host disease as a high-risk factor for osteoporosis (28) as the induced changes in the immune system lead to bone destruction. The inflammatory TNF signaling pathway was clearly expressed in the present analysis. A recent study revealed that tumor necrosis factor (TNF) directly enhances RANKL expression and promotes osteoclast formation (29).In bone metabolism (30), multiple target genes on the TNF pathway regulate osteoclast differentiation, thereby regulating physiological bone remodeling and participating in the pathogenesis of various bone diseases, such as osteoporosis and bone loss under inflammatory conditions. The ECM-receptor interaction pathway is mainly associated with cell communication, proliferation, and adhesion (31), At present, this pathway is primarily a marker for cancer and malignant tumors (32). However, a study (33)showed that the ECM-receptor interaction pathway may play a role in the impaired fracture healing of osteoporotic mice and is considered one of the causes of bone non-union (34).

Notably, multiple key genes associated with the circadian rhythm were significantly expressed in our results, including Arntl, Npas2, Dbp, Per1, and Per2. Our analysis confirmed the important role of the circadian rhythm in osteoporosis and the positive regulatory effect of Rhizoma Drynariae. Circadian rhythms are controlled by a molecular clock system located in the suprathalamic optic chiasmus (central clock) and the peripheral tissue (peripheral clock). Some studies have revealed that the disruption of circadian rhythms can lead to bone remodeling disorders and eventually osteoporosis because various bone regulatory growth factors are associated with clock genes (35). Fu (36) showed that the circadian rhythm is transmitted to peripheral osteoclasts and proved the role of clock genes in osteoclasts by extracting mRNA from the femur at different time periods and then analyzing the expression of osteoclast-related genes and clock genes. Osteoclast-related genes, such as Ctsk and Nfatc1, show distinct circadian rhythms.

Npas2, also known as neuronal PAS domain protein 2, is also recognized as a tumor suppressor (37), It has been shown that Npas2, as one of the core circadian rhythm genes, can play a role in cell development by regulating DNA-related genes. In one study, NPAS2 was found to show high expression in a human fibrosis specimen and in a mouse fibrosis model (38). It indicates the profibrotic role of Npas2. A study of Morinaga (39) showed, after the placement of titanium biomaterial in mice, mice were found to be in a state of accelerated bone integration, where NPAS2 expression was up regulated, revealing that NPAS2 may be a possible mechanism of action involved in bone metabolism as a circadian gene *in vivo*. NPAS2 usually cooperates with Arnt-1 (BMAL1) to regulate the transcription of circadian genes.BMAL1 also acts as a core component gene of circadian rhythms, and increasing evidence indicates that a key role of BMAL1 in bone development can promote fracture healing and improve bone. Animal experiments confirm that when BMAL1 receives inhibition, bone and cartilage development is hindered and thus caused by a decrease in bone mass (40).Bunger showed that the Bmal1-null mice exhibit reduced bone mineral density and muscle strength. Cytomolecular experiments found that BMAL1 exhibited a synergistic effect on the proliferation and differentiation of BMSC, and BMAL1 deficiency inhibited osteoblast and chondrocyte differentiation while promoting the differentiation and formation of osteoclasts and increasing bone resorption (41, 42).Therefore, our experiment suggests the mechanism of action of the circadian rhythm in osteoporosis and the positive regulatory effect of Rhizoma Drynariae.

Previous findings show that DBP in omental adipose tissue has low expression in subcutaneous adipose tissue in patients with type 2 diabetes mellitus(T2DM), confirming its involvement in regulating the expression of genes related to lipid metabolism (43).As the major carrier protein of vitamin D metabolites, DBP is able to maintain calcium homeostasis through the targeted transport of vitamin D. Current studies have shown that DBP can be activated to (DBP-MAF) and then directly activate osteoclasts to promote bone resorption (44).

In this analysis, it was found that many genes about fat metabolism were also significantly expressed in differential genes and enriched pathways. It shows a positive regulatory role of Rhizoma Drynariae in regulating osteogenic differentiation and fat differentiation. Gremlin-2, a key bone morphogenetic protein (GREM2) (BMP) antagonist, can competitively bind BMP receptors to regulate the activity of BMP ligands, thus it plays a crucial role in regulating bone homeostasis (45). In the Yu Feng’s study (46) showed that the GREM2 gene polymorphisms were positively associated with the risk of osteoporosis in postmenopausal women. It has been shown that GREM2 negatively regulates osteoblast differentiation by participating in the regulation of the Wnt/catenin signaling pathway (47). In the analysis of our experimental, the results also found that the significant regulation of GREM2 occurs with the involvement of Wnt/catenin.

Several genes related to lipid metabolism, such as HPGD and DNER, were also confirmed to be significantly regulated in this analysis. It shows that Rhizoma Drynariae can regulate proteins and pathways related to lipid metabolism, improve the anti-oxidative stress resistance, thus promoting osteogenic expression and reversing osteoporosis.

Lisa et al. (48) showed the PPAR-induced HPGD expression in visceral adipose tissue (VAT) Treg cells. The conditional deletion of Hpgd in murine Treg cells causes local inflammation and systemic insulin resistance. Patients with type 2 diabetes exhibit reduced HPGD expression in Treg cells. These data suggest that HPGD is likewise involved in adipose tissue homeostasis. Dner acts as a Notch and EGF-related receptor (49), playing an important role in the adipogenic differentiation of human adipose tissue-derived mesenchymal stem cells. Studies revealed that a specific siRNA knockdown of Dner, eventually leads to increased adipose differentiation (50, 51). Wang (52) showed that Positive correlation between DNER and Wnt/catenin signaling, We speculate that DNER also promotes osteogenesis. Previous studies (53) found that DNER expression was found to affect the expression of pathway genes in pancreatic cells, Reduce the occurrence of lipidation.

It also shows that osteoporosis is closely linked to T2DM. The AGE-RAGE signaling pathway is important in diabetes complications, including reduced bone mineral density and impaired bone quality. Our study confirmed that AGE-RAGE signaling has a role in promoting increased osteogenic function in bones (54), Moreover, the effects of Rhizoma Drynariae on the AGE-RAGE signaling pathway and lipid metabolism-related pathways have been extensively studied. In addition to T2DM, the diseases related to Rhizoma Drynariae regulation were also correlated with alcoholism and amoebiasis.

Although previous bioinformatics studies have been carried out on the effect of Rhizoma Drynariae in postmenopausal osteoporosis (15), no further verification has been conducted. In our DEG analysis, we have verified that Rhizoma Drynariaer in osteoporosis model rats occurs through multiple pathways and targets. However, this study is limited by its small sample size, the difficulty of RNA extraction from bone tissue, and some errors that may affect the experimental results to a certain extent. Although the mechanisms of Chinese herbal medicine in the treatment of osteoporosis have been widely studied, the lack of a more comprehensive analysis necessitates further research. In this regard, transcriptome analysis can broaden the scope of research and provide new directions to these studies.

## Data Availability Statement

The original contributions presented in the study are publicly available. This data can be found here: https://www.ncbi.nlm.nih.gov/bioproject/PRJNA862708, Accession: PRJNA862708; ID: 862708.

## Ethics Statement

The animal study was reviewed and approved by Affiliate Hospital of Shandong University. Written informed consent was obtained from the owners for the participation of their animals in this study.

## Author Contributions

ZX, GT, and HS designed and developed the experiments. HS and HX prepared the draft of the manuscript. HS, HX, GT, ZX, SG, BY and RW participated in all the experiments. HS, BY, RW and HX analyzed the data and drafted the manuscript.HX, GT, LZ, and ZX supervised all research and revised the manuscript. All authors contributed to the article and approved the submitted version.

## Funding

This study was funded by National Natural Science Foundation of China (82174410); Natural Science Foundation of Shandong Province, ZR2020KH011 Natural Science Foundation of Shandong Province, ZR2020MH362.

## Conflict of Interest

The authors declare that the research was conducted in the absence of any commercial or financial relationships that could be construed as a potential conflict of interest.

## Publisher’s Note

All claims expressed in this article are solely those of the authors and do not necessarily represent those of their affiliated organizations, or those of the publisher, the editors and the reviewers. Any product that may be evaluated in this article, or claim that may be made by its manufacturer, is not guaranteed or endorsed by the publisher.

## Abbreviations

CT, computerized tomography; DEG, differentially expressed gene; ECM, extracellular matrix; H&E, hematoxylin and eosin; GO, gene ontology; KEGG, Kyoto Encyclopedia of Genes and Genomes; PPI, protein-protein interaction; STRING, Search Tool for the Retrieval of Interacting Genes; T2DM, type 2 diabetes.
